# NF-κB Induced the Donor Liver Cold Preservation Related Acute Lung Injury in Rat Liver Transplantation Model

**DOI:** 10.1371/journal.pone.0024960

**Published:** 2011-09-16

**Authors:** An Jiang, Chang Liu, Yulong Song, Feng Liu, Quanyuan Li, Zheng Wu, Liang Yu, Yi Lv

**Affiliations:** 1 Department of Hepatobiliary Surgery, the First Affiliated Hospital, School of Medicine, Xi'an Jiaotong University, Xi'an, China; 2 Department of Anesthesiology, Renmin Hospital of Shaanxi Province, Xi'an, China; 3 Department of Liver Transplantation, Qianfoshan Hospital, Jinan, China; 4 Department of Transplantation, Dongfeng Hospital, Shiyan, China; University of Colorado Denver, United States of America

## Abstract

**Background:**

We have observed at our clinical work that acute lung injury (ALI) often occurs in patients transplanted with donor livers persevered for long time. So, we conducted this study to investigate the influence of cold preservation time (CPT) of donor liver on ALI induced by liver transplantation (LT), and further study the role of nuclear factor-κB (NF-κB) in the process.

**Methods:**

Wistar rats were used as donors and recipients to establish orthotopic rat liver transplantation models. Donor livers were preserved at 4°C for different lengths of time. The effect of NF-κB inhibitor, ammonium pyrrolidinedithiocarbamate (PDTC), on ALI was detected. All samples were harvested after 3 h reperfusion. The severity of liver injury was evaluated first. The expressions of tumor necrosis factor-α (TNF-α) and interleukin-1β (IL-1β) in liver tissue and liver outflow serum were measured respectively. The severity indexes of ALI, the activity of NF-κB and inhibitor-κBα (I-κBα) in lung/liver were measured accordingly.

**Results:**

With the prolonged liver CPT, the liver damage associated indexes and ALI-related indexes all increased significantly. TNF-α and IL-1β in liver outflow serum increased accordingly, and the activity of NF-κB in liver/lung increased correspondingly. All these ALI-associated indexes could be partially reversed by the use of PDTC.

**Conclusions:**

Extended CPT aggravates the damage of donor liver and induces the expressions of TNF-α and IL-1β in liver. These inflammatory factors migrate to lung via liver outflow blood and activate NF-κB in lung, inducing ALI finally. NF-κB may play a critical role in LT-related ALI. Patients with or at risk of ALI may benefit from acute anti-inflammatory treatment with PDTC.

## Introduction

The incidence of acute lung injury (ALI) post liver transplantation (LT) has been reported to be 60–80% [1]. ALI may develop into its severest form, acute respiratory distress syndrome (ARDS), and the morbidity rate of ARDS was reported to be 4.5%–18%, and the case fatality rate was 50%–70% [1], [2]. Therefore, investigating the etiological factors and further preventing the complications is important in reducing in-hospital mortality post LT [3]. Intraoperative transfuse plasma-containing blood products (red blood cells, platelet, etc.) are involved in the development of ALI post LT, which is called transfusion-related acute lung injury [4], [5]. And we have observed at our clinical work that ALI often occurs in patients transplanted with donor livers persevered for long time, but the relationship between cold preservation time (CPT) of donor liver and the occurrence of ALI was not very clear.

Nuclear factor-κB (NF-κB) is a critical intracellular mediator of the inflammatory cascade, which can trigger the expression of multiple inflammatory genes including tumor necrosis factor-α (TNF-α) and interleukin-1β (IL-1β) [6]. Ammonium pyrrolidine dithiocarbamate (PDTC) is a potent inhibitor of NF-κB. Recent studies have shown that NF-κB plays an essential role in the regulation of genes whose products are involved in the pathogenesis of hepatic cold preservation/warm reperfusion (CP/WR) injury and LT-induced ALI [7], [8]. Therefore, we raised a hypothesis that prolonged CPT of donor liver might induce liver damage, and the inflammatory factors in liver would migrate to lung, activate the NF-κB in lung, and in turn induce ALI. The purpose of this study was to assess the relationship between donor liver CPT and LT-related ALI, and further to study the role of NF-κB in the process.

## Materials and Methods

### Experimental design

Male Wistar rats (n = 110) were used to establish orthotopic liver transplantation models. To avoid allograft rejection interference in the process of LT-related ALI, we used the rats as both donors and recipients. The rats were randomly divided into 6 groups according to CPT of the donor livers (n = 10 in sham group, n = 20 in 5 LT groups). Donor livers were preserved in 4°C University of Wisconsin (UW) solution respectively for 0 min (sham operation), 45 min, 90 min, 120 min, 180 min, and 180 min plus intravenous injection of PDTC at a dosage of 100 mg/kg after liver reperfusion [9]. All recipients were sacrificed at 180 min after LT.

### Animal preparation and sample collection

A total number of 110 adult male Wistar rats (272±31 g; Tongji Medical Center, Central China University of Science and Technology) were used as donors and recipients. Prior to the experiment, the rats were fasted for 12 h and allowed free access to water. Liver harvesting and LT were performed under anesthetization by intraperitoneal injection of ketamine hydrochloride (80 mg/kg). LT was conducted using the method described by Kamada and Calne [10]. Protocols for animal care and experimental management were approved by the Xi'an Jiaotong University Animal Experimentation Committee (Approval Number: XJTU2007-43) and performed according to the Xi'an Jiaotong University Guidelines for Animal Experimentation.

The hepatic venous outflow blood was sampled using a venous retention needle. The suprahepatic vena cava was punctured first, the soft needles were pushed into the right hepatic vein which is relatively thick in rat, and the blood was slowly collected. The blood samples were centrifuged immediately to isolate serum for determining the serum levels of TNF-α, IL-1β and Malondialdehyde (MDA). Part of the left lower lobe of each lung was fixed with polyoxymethylene for histological examination. Lung tissue homogenate (10%) was made with 200 mg left upper lobe of lung and 1.8 ml phosphate-buffered saline (PBS), then stored at −80°C for the analysis of myeloperoxidase (MPO). The rest lung tissue was immediately frozen in liquid nitrogen and kept at −80°C until use.

### Analysis of serum MDA

Serum MDA level was measured by using MDA assay kit (Nanjing Jiancheng Bioengineering Institute, China) according to the manufacturer's instructions. Then the absorbance of the reaction mixture was read at 532 nm in a microplate reader (Bio-Tek Instruments Inc., USA). The data were expressed in nmol/L.

### Measurement of alanine transarninase (ALT) and aspartate aminotransferase (AST) in serum

Serum levels of ALT and AST were measured using a Hitachi 7160 analyzer (Hitachi Inc., Japan) and expressed in IU/L.

### Measurement of apoptosis index (AI) in liver and lung

AI in liver and lung were assessed by terminal deoxynucleotidyl transferase mediated dUTP nick end-labeling (TUNEL) assay using DeadEnd™ Colorimetric Apoptosis Detection Kit (Promega Inc., USA), following the procedures described in the manufacturer's protocol. Cells were considered TUNEL-positive when nuclear staining was intense, dark brown and homogeneous. The TUNEL-positive cells were counted in 6 random microscopic fields under the light microscope at a magnification of 400. Approximately 1000 pneumonocytes were examined in each high power field. The AI was defined as the number of stained cells per high power field.

### Measurement of TNF-α and IL-1β in liver and NF-κB P65 in liver and lung

Histological sections (4 µm) were cut on a rotary microtome and stained to detect the intragraft expression of TNF-α and IL-1β, as well as NF-κB P65 in liver and lung. Paraffin sections were spread on a slide, and rabbit anti-rat TNF-α, IL-1β and NF-κB P65 polyclone antibodies (diluted 1∶200, Santa Curz Corp., USA) were used to detect the intragraft expression of TNF-α, IL-1β and NF-κB P65, respectively. Biotin-labeled goat anti-rabbit secondary antibody (Santa Curz Corp., USA), HRP-labeled anti-biotin, and 3, 3-diaminobenzidine were used to visualize the positive expression. We counted 10 randomly chosen fields per section using a light microscope at high power (400 magnification; Q550CW Leica Corp., Germany) and the results were expressed as absorbance unit (AU).

### Quantitation of serum TNF-α and IL-1β

Serum levels of TNF-α and IL-1β were determined using a commercial enzyme-linked immunosorbent assay (ELISA) kit (Jingmei Biotech Corp., China). All reagents, samples and standards were prepared according to the manual of the kit. After ELISA reaction, the absorbance was measured using a microwell-plate reader (Bio-Tek Instruments Inc., USA) at 450 nm.

### Measurement of NF-κB DNA binding activity

NF-κB DNA binding activity was measured by electrophoretic mobility shift assay (EMSA) as described in Jung et al. [11]. Briefly, tissue nuclear extracts (5 µg) were incubated for 20 min on ice in binding reactions with radio-labeled DNA probes containing the consensus binding sites for NF-κB, and then subjected to nondenaturing polyacrylamide gel electrophoresis. A shift in mobility corresponding to protein binding was assessed by autoradiography and quantitated by phosphor imager analysis.

### Preparation of nuclear and cytoplasmic subcellular fractionation

Frozen lung tissue of 100 mg was homogenized with 5 ml of cytoplasmic extraction buffer [150 mmol NaCl, 10 mmol Hepes (pH 7.9), 1 mmol EDTA, 0.5 mmol PMSF, 1 mmol MgCl_2_, 1 mmol DTT, 3 mg/l Leupeptin]. After 15 min incubation on ice, the cellular suspension was centrifuged at 5,000 rpm for 15 min at 0°C. The supernatant, representing the cytoplasmicic fractions, was removed. The nuclear pellet was thawed on ice for 20 min and suspended in 500 µl of nuclear extraction buffer [25% Glycerol, 20 mmol Hepes (pH 7.9), 420 mmol NaCl, 1.2 mmol MgCl_2_, 5 mg/l Pepstatin, 5 mg/l Leupeptin, 0.5 mmol DTT, 0.5 mmol EDTA, 0.5 mmol PMS], incubated at 0°C for 20 min, and centrifuged at 14,000 rpm for 20 min at 4°C. And the supernatant, representing the nuclear fraction now, was removed. After added with 0.2% protease inhibitor cocktail (Roche Corp., Switzerland), these fractions were stored at −80°C.

### Measurement of NF-κB and I-κBα

The levels of NF-κB and I-κBα were measured by western blot analysis. Nuclear and cytoplasmic subcellular fractionation was denatured in loading buffer and was electrophoresed on a 10% SDS-polyacrylamide gel (SDS-PAGE). After the completion of electrophoresis, the proteins were transferred to PVDF membrane filters (Millipore Biotechnology Inc, USA). The transferred membranes were incubated overnight at −4°C with primary rabbit polyclonal anti-NF-κB P65 antibody and rabbit polyclonal anti- IkappaB Kinase (IkB)-α antibody (1∶500 dilutions; Santa Cruz Biotechnology Inc., USA) in PBS-T that contained 5% skim milk. After washing four times in PBS-T, the membranes were incubated with anti-rabbit immunoglobulin G conjugated to horseradish peroxidase (Wuhan Boster Biotechnology Inc., China) at a dilution of 1∶5000 in PBS-T for 1 h at room temperature. After four additional washes with PBS-T, the signals were visualized by Super Signal detection systems (Millipore Biotechnology Inc., USA) and exposed to film. Histone and β-actin were used as internal controls to normalize the expressions of NF-κB and I-κBα.

### Measurement of lung homogenate MPO

Fresh lung tissues were extracted, following the homogenization procedures described in the manufacturer's protocol. The level of MPO in lung tissue was measured by ELISA kit (Nanjing Jiancheng Bioengineering Institute, China). The absorbance was measured using a microwell-plate reader (Bio-Tek Instruments, Inc., USA).

### Quantitation of lung permeation index (LPI)

Lung lavage fluid was obtained by irrigating the lung with 2 mL PBS. Total protein concentrations in lung lavage fluid and in serum were measured by the BCA protein quantitation kit (Pierce Biotechnology, USA). The results were expressed as LPI (protein concentration in lung lavage fluid/protein concentration in serum).

### Data analysis

All the data were represented as mean±standard deviation and analyzed with one-way analysis of variance and student *t*-test. All the statistical analyses were carried out via SPSS 13.0 and *P*<0.05 were considered to be statistically significant.

## Results

### General conditions of the animals

The duration of donor operation was 45.6±13.8 min, the time for donor liver preparation was 20.3±6.7 min, and warm ischemia was avoided. The receptor operation lasted for 26.7±5.5 min, and the anhepatic phase lasted for 16.1±2.5 min. No significant difference was seen in portal clamping time among the groups.

### Serum MDA, ALT and AST levels increased with prolonged CPT

**Figure 1:**
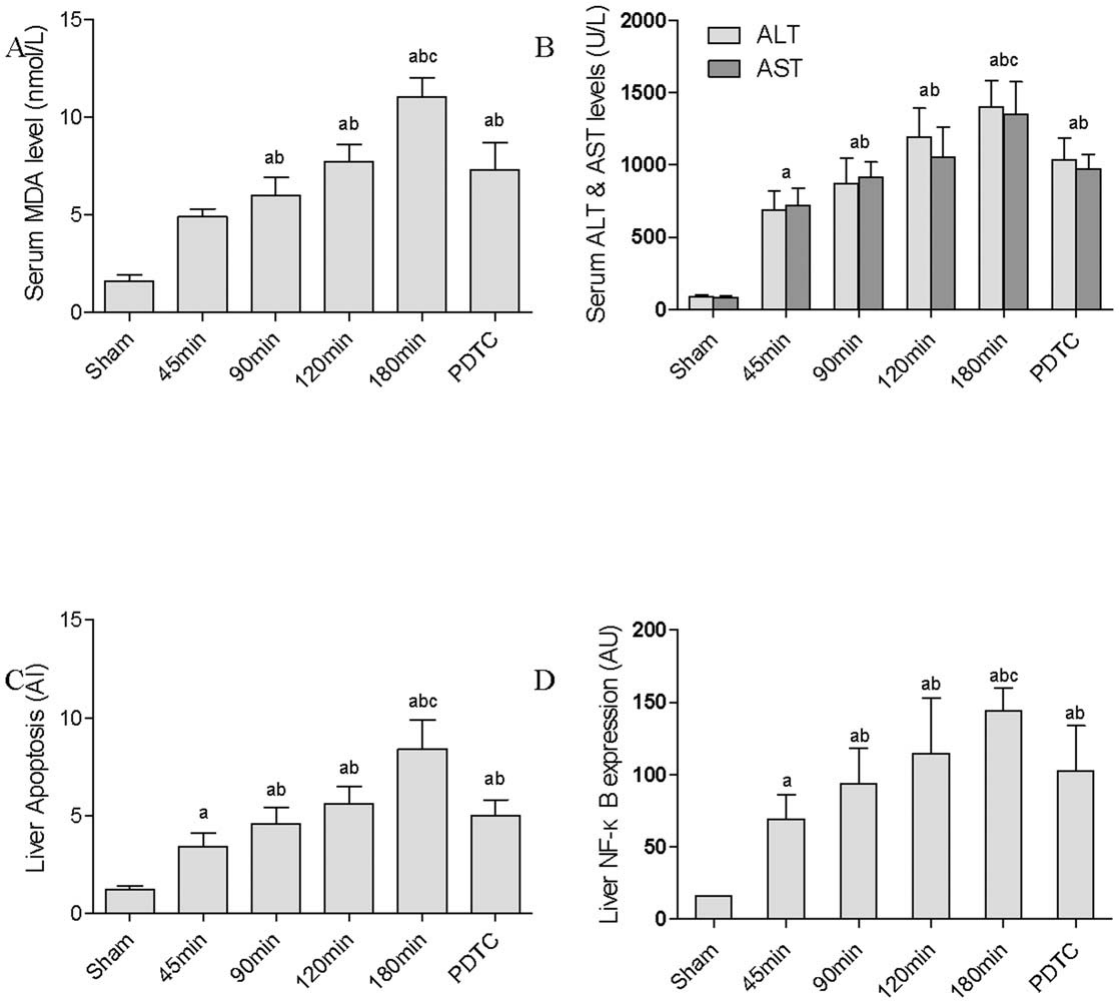
The liver injury indexes post rat LT with different donor liver CPT. (A) Serum MDA level. (B) Serum ALT and AST levels. (C) Liver apoptosis index (AI). (D) NF-κB P65 expression in liver. The results were expressed as absorbance unit (AU). (^a^ *P*<0.05 *vs.* sham group, ^b^ *P*<0.05 *vs.* 45 min group, ^c^ *P*<0.05 *vs.* PDTC group).

The levels of serum MDA (a lipid peroxidation marker), ALT and AST (hepatocytes injury markers) increased significantly with longer liver CPT. There was statistical difference between each of the operation groups and the control group (*P*<0.05, 45 min group, 90 min group, 120 min group and 180 min group *vs.* sham group). The expressions of MDA, ALT and AST were inhibited significantly by the use of PDTC (*P*<0.05, 180 min group *vs.* PDTC group, Fig. 1A, B).

### Hepatocytes apoptosis increased with prolonged CPT

**Figure 2:**
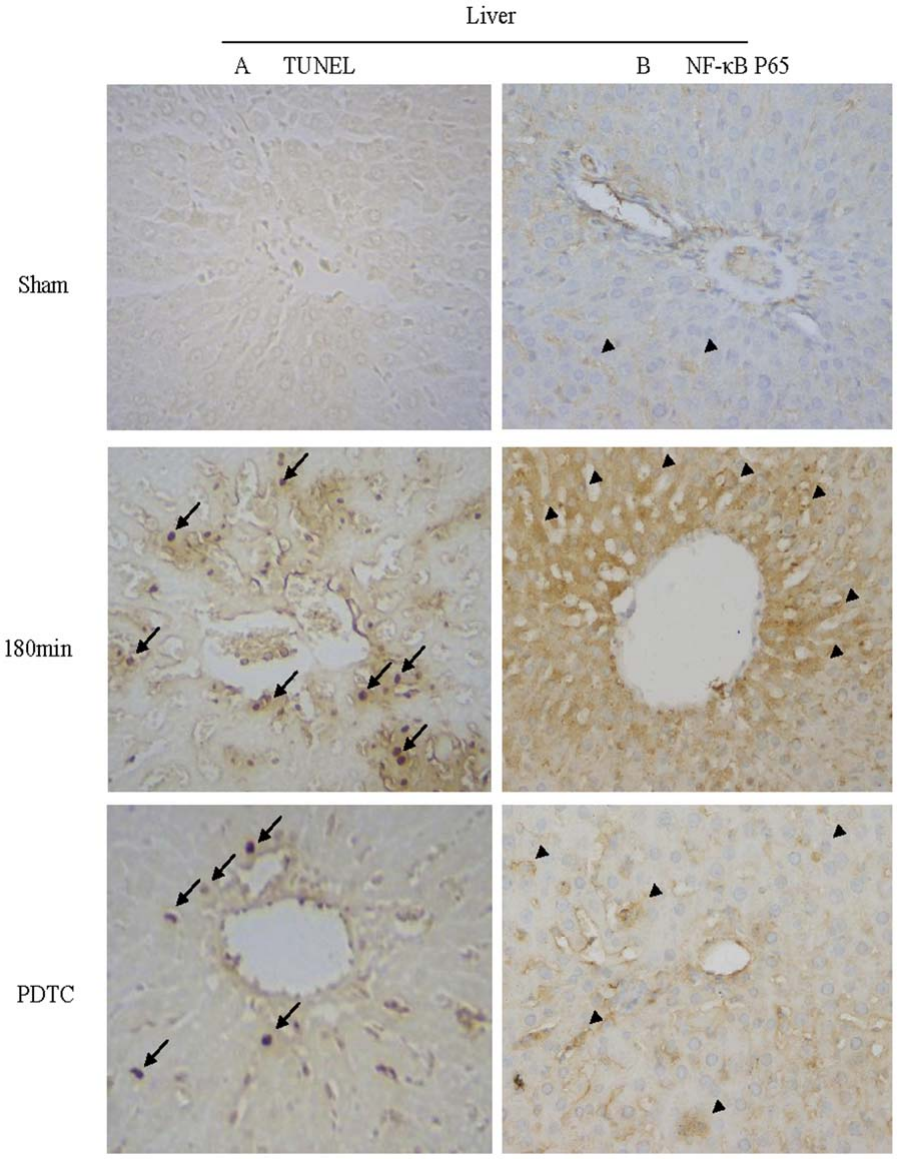
Representative tissue sections of liver apoptosis and NF-κB P65 expression (×400). (A) TUNEL-stained liver tissues with different CPT. TUNEL-positive cells are revealed by brown stain (indicated by arrows). (B) Liver immunohistochemistry sections of NF-κB P65 with different CPT. Positive stain was homogeneously distributed in liver sections (indicated by arrow heads).

The mean AI of hepatocytes in all the 5 operation groups significantly increased compared with that in the sham group (*P*<0.05), and the increase was found to be time-dependent. The AI in the PDTC group was lower than that in the 180 min group (*P*<0.05, Fig. 1C). TUNEL-positive hepatocytes were revealed by brown stain, and most were located in hepatic lobule (Fig. 2A). These results suggested that with the CPT prolonged, the CP/WR injury of donor liver got more severe,and more hepatocytes underwent apoptosis.

### Liver NF-κB P65 expression increased with prolonged CPT

The concentration of liver NF-κB P65 was determined by immunohistology. With prolonged CPT, the expression of NF-κB P65 displayed a significant increase in almost all the LT groups (*P*<0.05) but the PDTC group, which indicated that the NF-κB P65 expression level was inhibited after the intravenous injection of PDTC (*P*<0.05, Fig. 1D). The positive stain was homogeneously distributed in liver sections (Fig. 2B). The results suggested that NF-κB was involved in CP/WR-induced liver injury.

### TNF-α and IL-1β levels in donor liver and liver outflow blood serum increased with prolonged CPT

**Figure 3:**
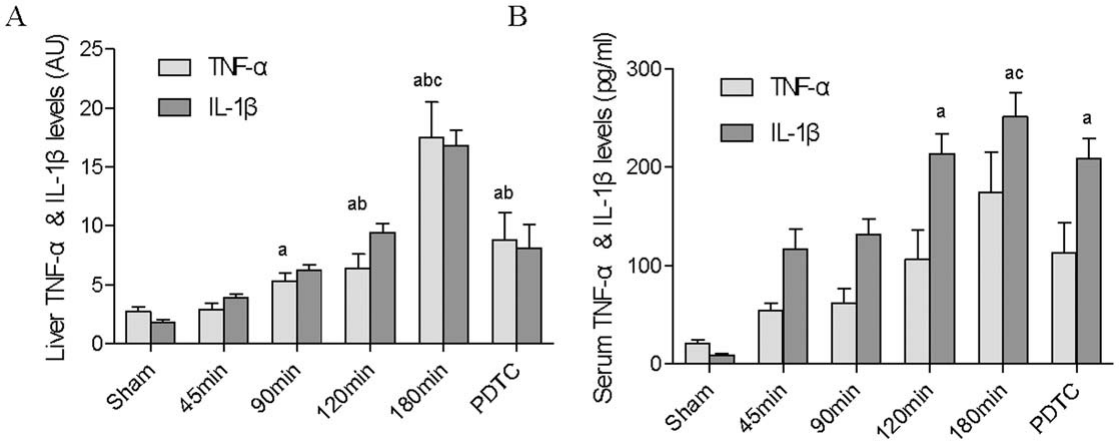
The expressions of TNF-α and IL-1β in donor liver and liver outflow blood serum. (A) TNF-α and IL-1β levels in donor liver by immunohistochemistry. The results were expressed as absorbance unit (AU). (B) TNF-α and IL-1β expressions in the liver outflow blood serum detected by ELISA. (^a^ *P*<0.05 *vs.* sham group, ^b^ *P*<0.05 *vs.* 45 min group, ^c^ *P*<0.05 *vs.* PDTC group).

Fig. 3A and 3B show that with prolonged CPT, TNF-α and IL-1β in liver and liver outflow blood serum increased significantly (*P*<0.05). After administration of PDTC, TNF-α and IL-1β production was significantly attenuated (*P*<0.05). It was suggested that the activated NF-κB in liver induced some downstream target genes such as TNF-α and IL-1β. These inflammatory factors produced in donor liver and transported to lung through liver outflow blood stream.

### DNA binding activity of NF-κB in lung increased with prolonged CPT

**Figure 4:**
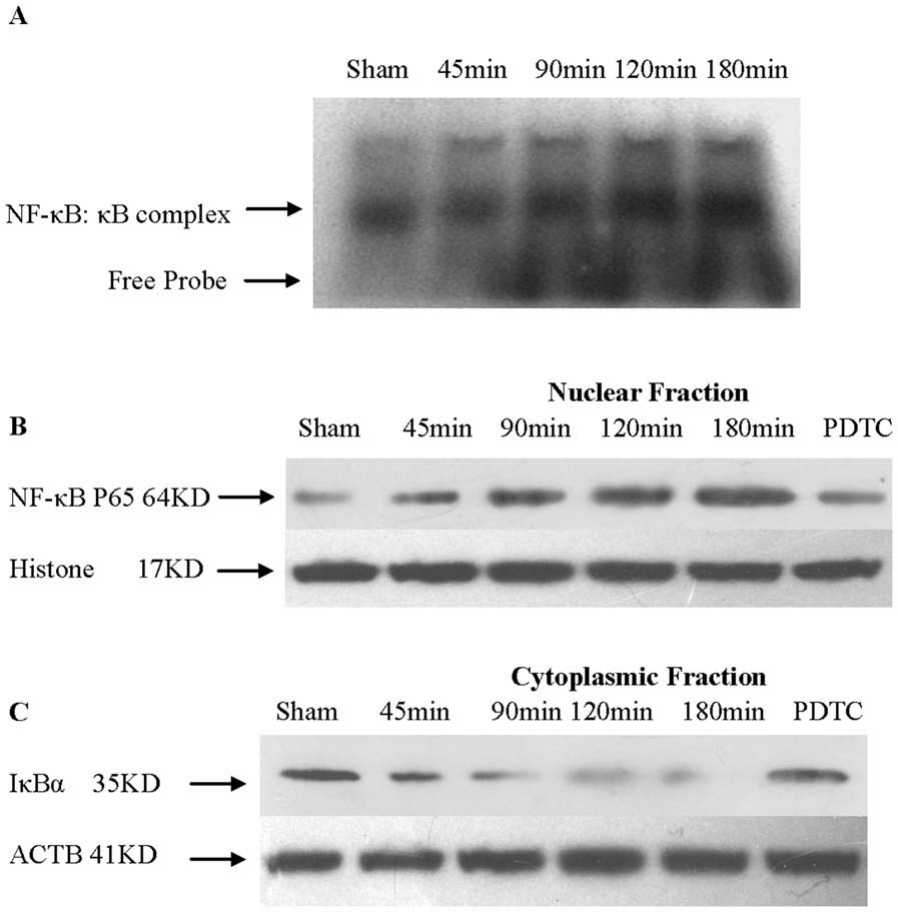
The DNA binding activity of NF-κB, NF-κB P65 localized in nucleus, and I-κBα degraded in cytoplasmic with prolonged CPT. (**A**) **The DNA binding activity of NF-κB.** Lung tissue extracts were incubated with a ^32^P-labeled κB DNA probe, and the DNA binding activities were analyzed by EMSA. Arrows indicate the position of NF-κB: kB DNA complex, and nonspecific DNA bindings. (B) Level of NF-κB P65 localized in nucleus. The NF-κB P65 levels increased with prolonged CPT and was inhibited by PDTC. (C) The level of I-κBα in cytoplasmic subcellular. I-κBα fractions were degraded with prolonged CPT, and protected by the use of PDTC.

The NF-κB DNA binding activity in lung tissue extracts was assessed by EMSA using an oligomer containing a consensus NF-κB site. As shown in Fig. 4A, the DNA binding activity of NF-κB in lung tissue increased significantly with pronged CPT.

### NF-κB P65 localized in nucleus with prolonged CPT

The lung tissue was prepared using a subcellular fractionation method to isolate the nuclear and cytoplasmic cellular proteins. Western blot analysis showed an increase in NF-κB nuclear protein in response to prolonged CPT, which reached a maximum at the CPT of 180 min. In contrast, treatment with PDTC post transplantation prevented NF-κB nuclear localization (Fig. 4B). The results suggested that donor liver CP/WR injury increased signal transduction pathway of NF-κB in lung, and translocated NF-κB into nucleus.

### Liver cold preservation induced degradation of cytoplasmic I-κBα in lung

Through a protein-protein interaction, I-κBα blocks the NF-κB nuclear localization sequence, thus preventing the transport of NF-κB into the nucleus. To determine whether I-κBα was degraded by the prolongation of liver CPT, lung tissue cytoplasmic I-κBα protein levels were examined at different CPT via western blot analysis. With prolonged donor liver CPT, the I-κBα protein levels decreased significantly to almost undetectable level in the 180 min group. After the use of PDTC, I-κBα returned to relatively higher levels (Fig. 4C). These results suggested that NF-κB inhibitor interfered with the degradation of I-κBα, thereby preserving the subsequent nuclear localization of NF-κB.

### CPT-induced NF-κB P65 expressed mainly in alveolar epithelial cells

**Figure 5:**
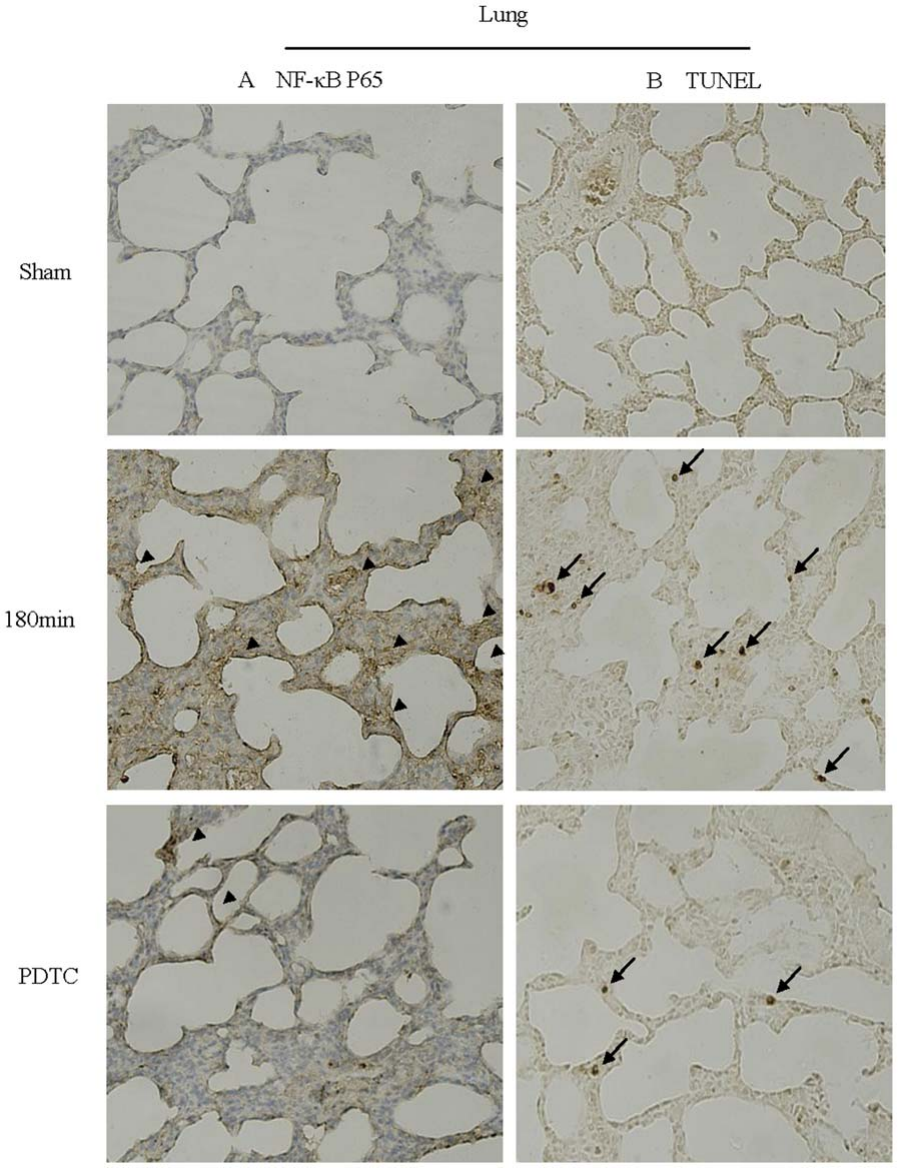
Representative tissue sections of lung apoptosis and NF-κB P65 expression(×400). (A) Immunohistochemistry stains of NF-κB P65 in lung with different CPT. Brown stain indicates positive cells (arrow head). (B) TUNEL-stained lung tissues with different CPT. These slices demonstrate that the positive signals increased with prolonged CPT and decreased after the use of PDTC. Arrows identify representative TUNEL-positive cells.

It was found that immunostaining of NF-κB P65 in lung increased with extended CPT. Morphological observation of the tissue section showed that most positive signals were located in alveolar epithelial cells, and NF-κB p65 positive signals mainly expressed in nucleus (Fig. 5A). The positive expression of NF-κB P65 in lung tissue decreased in the PDTC group, which suggested that the use of PDTC inhibited the expression of NF-κB.

### Pneumonocyte apoptosis increased with prolonged CPT

**Figure 6:**
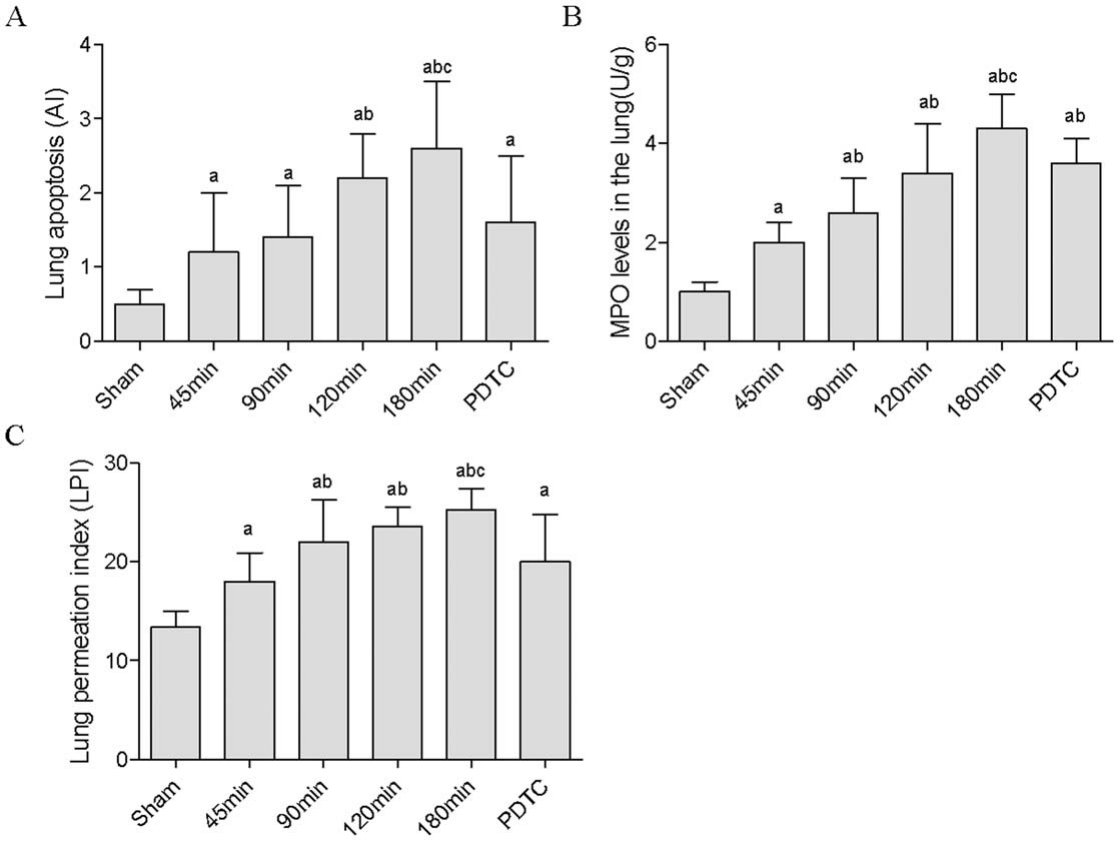
ALI-related indexes with different CPT. (A) Apoptosis Index (AI) of lung with different CPT. (B) MPO expression levels with prolonged CPT. (C) Lung permeation index (LPI) with different CPT. (^a^ *P*<0.05 *vs.* sham group, ^b^ *P*<0.05 *vs.* 45 min group, ^c^ *P*<0.05 *vs.* PDTC group).

The degree of apoptosis in lung tissue was determined by TUNEL staining (Fig. 5B). AI increased when CPT was prolonged. There was statistical difference between the 120 min group and 180 min group versus sham operation group (*P*<0.05), and 120 min group and 180 min group versus 45 min group (*P*<0.05), respectively. AIs decreased when NF-κB inhibitor was used (*P*<0.05, Fig. 6A).

### Liver cold preservation increased MPO expression

MPO has been used as a marker of pulmonary neutrophil accumulation and activation. In our experiment, the expression of MPO in lung increased with prolonged CPT, and there was statistical difference between each of the LT groups and sham group (*P*<0.05). The level of MPO decreased when PDTC was used (*P*<0.05, Fig. 6B).

### LPI increased with prolonged CPT

With the prolongation of CPT, LPI increased significantly. In comparison with that of the 45 min group, LPI of other LT groups increased significantly (*P*<0.05). But in the PDTC group, LPI decreased remarkably (*P*<0.05, Fig. 6C). These results indicated that with prolonged CPT, lung permeation which was induced by pneumonocyte apoptosis increased, and this in turn aggravated the pathogenesis of ALI.

## Discussion

NF-κB is one of core transcription factors, which triggers multiple inflammatory genes including TNF-α, IL-1β and various adhesion molecules. In cytoplasm, NF-κB exists as an inactive form bound with I-κBα. After an injury, a specific IκB-kinase complex phosphorylates and degrades I-κBα by the proteasome. The I-κBα degradation allows NF-κB to translocate into nucleus where it acts as a transcription factor for the generation of inflammation factors (TNF-α and IL-1β, etc.). Many studies have demonstrated that the antioxidant PDTC represents a specific potent inhibitor of NF-κB. In addition, PTDC blocks the phosphorylation of I-κBα, the dissociation of NF-κB from I-κBα, and the subsequent translocation of NF-κB to the nucleus in response to inflammation [9].

ALI is a common complication post LT [12]. Despite intense research and diverse therapeutic trials, there is no effective prevention or treatment for LT-related ALI at present [7]. ALI is caused by various factors and is characterized by the pathology of pulmonary alveolus injury. The hallmark of ALI/ARDS is diffuse alveolar damage and increased pulmonary microvascular permeability with increased protein content of the edema fluid [5].

The pathogenesis of ALI involves the disorders of oxidant/anti-oxidant and inflammation/anti-inflammation [13]. Guo et al. analyzed the serum cytokine expression in LT-related ALI patients by using RayBio human antibody array, and identified changes in expression of various inflammatory factors [14]. NF-κB, as a critical intracellular mediator of the inflammatory cascade, is a notable contributory factor in ALI. Therefore, we take NF-κB into consideration in our investigation of LT-related ALI induced by donor liver CP/WR injury.

Our findings that the levels of MDA, ALT, AST and AI increased with the prolonged CPT indicate that the donor liver suffers more severe CP/WR injury with longer CPT. The activity of NF-κB in donor liver increased, which enhanced the transcription of TNF-α and IL-1β in liver. The hepatic production of TNF-α, a central mediator in the hepatic response to CP/WR injury, could increase shortly after reperfusion and had powerful effects on not only the local hepatic environment but also remote organ function, most notably in lung [15]. We have found that in liver outflow blood, the serum TNF-α and IL-1β levels both increase; consequently, more TNF-α and IL-1β flow in lung via liver outflow stream. The increase of serum concentrations of TNF-α and IL-1β in ALI patients post LT has also been reported [16].

NF-κB in lung is activated by the inflammation factors in blood. At the same time, the level of I-κB decreases accordingly. This process induces more aggressive inflammation reaction in lung and up regulates the ALI-related indexes including levels of LPI, lung AI and lung MPO, as a result of which, ALI finally occurs. On the other side, all these factors can be partly reversed by the NF-κB inhibitor PDTC, which reveals that NF-κB should be an important factor in the process.

A large number of studies demonstrate that multiple cell apoptosis occurs in ALI. The incidence of ALI is mainly accompanied with abnormal apoptosis and excessive NF-κB activation [17]. The apoptosis of pulmonary alveolus endothelial cells and vascular endothelial cells directly leads to increased permeability and pulmonary edema [18]. We have found in our experiment that NF-κB P65 expresses mostly in alveolar epithelial cells, and the inflammatory injury of the cells contributes to ALI. Since the prolongation of liver CPT causes apoptosis of alveolar epithelial cells, increased apoptosis might be another important cause of ALI.

On the whole, with the prolongation of CPT, inflammatory factors in donor liver release to pulmonary circulation and activate NF-κB in lung; at the same time, the inflammatory factors induce the apoptosis of pneumonocytes, increase lung damage and cause ALI post LT. Furthermore, the cytokine storm, in which there is abnormal regulation of various cytokines, may occurred in post-LT patients with prolonged CPT, and could induce systemic inflammatory reaction syndrome, and in turn result in many organ injury including lung. Patients with or at risk of ALI may benefit from some anti-inflammatory treatment.
